# Editorial: Advances in cereals and millets nutrition research

**DOI:** 10.3389/fnut.2024.1349757

**Published:** 2024-04-10

**Authors:** Marta Mesías, Francisco J. Morales, Om Prakash Gupta, Harpreet Kaur Oberoi, K. N. Ganapathy

**Affiliations:** ^1^Institute of Food Science, Technology and Nutrition (ICTAN), Spanish National Research Council (CSIC), Madrid, Spain; ^2^Indian Council of Agricultural Research (ICAR)-Indian Institute of Wheat and Barley Research, Karnal, India; ^3^Punjab Agricultural University, Ludhiana, India; ^4^Indian Council of Agricultural Research (ICAR)-Indian Institute of Millets Research (IIMR), Hyderabad, India

**Keywords:** cereals, wheat, sorghum, millet, biofortification, nutrition, health

According to the latest FAO report on the state of food security and nutrition in the world (1), more than 720 million people faced hunger, and around 3 billion people did not have access to a healthy diet. All these problematics, exacerbated by the past COVID-19 crisis, led to an increase in the number of people affected by the so-called hidden hunger, caused by an inadequate intake of essential micronutrients (MNs) such as iron (Fe), zinc (Zn), selenium (Se) and provitamin A. Cereal grains constitute over 50% of the global population's primary caloric and protein intake, containing macronutrients (starch, protein, and lipids) and micronutrients (minerals and vitamins) crucial for nutritional value. Cereals also contain phytochemicals such as polyphenols, which have antioxidant and anti-inflammatory activity, although their bio accessibility depends on the type of polyphenol or cereal matrix involved (2). Despite significant yield improvements in cereal crops, essential nutrients for human health are often lacking, necessitating compositional enhancements to combat malnutrition (3). In the pursuit of nutrition-sensitive agriculture and sustainable food systems, a key goal is to improve the nutritional quality of cereals grains. With rising healthcare costs and an increase in lifestyle diseases such as obesity, diabetes, and cardiovascular issues, there is heightened scrutiny on the nutritional content of foods accessible to consumers and on the functionality and bioactivity of certain constituents. In this context, assessing the impact of processing on nutritional integrity and the preservation of health-beneficial bioactive compounds becomes crucial, recognizing that while processing plays a pivotal role in enhancing food, it may also have adverse effects (2).

During the last decade, there has been a renewed commitment to improve the nutritional value of major cereals and millets. This involves a comprehensive approach that includes an enhanced understanding of genetic diversity for grain nutrients in the germplasm collections, crop improvement efforts such as biofortification using conventional and advanced genomic approaches, advancements in food processing, value addition, considerations of nutrient bioavailability, and rigorous testing for food safety and quality.

Nutritionists have consistently emphasized the importance of dietary diversity as a fundamental component of high-quality nutrition. Millets are incredibly diverse, ancestral crops that are cultivated in various adverse climates and arid regions, have the potential to strengthen food security, and can contribute to a healthy diet due to their high nutritional value. Millets also known as nutricereals encompass a diverse group of dryland cereals, including pearl millet, sorghum, finger millet, proso millet, foxtail millet, barnyard millet, little millet, kodo millet, browntop millet and also includes fonio, teff and Job's tears. Millets are indigenous to many parts of the world, and form an intrinsic part of traditional culinary cultures, especially in India and sub-Saharan Africa with largest production. Although millets were one of the first domesticated crops, they are not widely known and their role in food security and local cultures often goes unrecognized. For this reason, the United Nations General Assembly declared 2023 as the International Year of Millets (IYM), based on the proposal by Government of India. The International Year of millets is a call to action, a moment to shed light on these neglected and underutilized crops, to amplify their many contributions and health benefits, and to create innovative market opportunities for many countries to benefit farmers and consumers. The IYM is also contributing to the UN 2030 Agenda for Sustainable Development, particularly SDG 2 (Zero Hunger), SDG 3 (Good health and wellbeing), SDG 8 (Decent work and economic growth), SDG 12 (Responsible consumption and production), SDG 13 (Climate action) and SDG 15 (Life on land). Millets are good source of protein, fiber, key vitamins, and minerals. The broad potential health benefits of millets include protecting cardiovascular health, preventing the onset of diabetes, helping people achieve and maintain a healthy weight, and managing a healthy gut ecosystem.

This Research Topic assembled five quality research papers, addressing various issues related to cereals and millets. The aim is to contribute to the expansion of research on grains as essential sources of nutrients in the diet. Some of the papers included have focused on sorghum and millet, illustrating how the biofortification of these cereals could give rise to nutritionally enriched grains. In the study of Nagesh Kumar et al. the focus is on the effective utilization of Indian landraces as a viable, cost-effective, and environmentally friendly approach in sorghum genetic biofortification. This approach aims to enhance sorghum productivity and nutritional content. Another paper by Yan et al. analyzed physicochemical and structural properties of different types of sorghum starches, providing insights for selecting and breeding sorghum varieties suitable for applications in both food and non-food industries. Regarding millet, Arora et al. contribute valuable information by outlining the nutrient profiles of various millet-based value-added products. Their findings suggest that incorporating these foods into the population's diets could play a significant role in preventing malnutrition and type 2 diabetes.

The importance that the biofortification is gaining globally to improve human nutrition through enhancing the micronutrient content in staple food crops has been mentioned in the manuscript of Manjunath et al.. In their study, the aim was to identify the chromosomal regions governing the grain iron concentration (GFeC), grain zinc concentration (GZnC), and thousand kernel weight (TKW) using recombinant inbred lines (RILs) in wheat, developed from a cross between HD3086 and HI1500. The study's results successfully identified specific molecular markers that can be used in practical plant breeding to develop biofortified varieties after their successful validation. In wheat, Li et al. investigated the formation of grain color and analyzed the correlated genes in various wheat lines at maturity. The outcomes of this research holds promise for establishing a theoretical foundation for understanding the processes involved in grain color formation during maturity. Moreover, the findings may shed light on the nutritional implications and the potential for product development associated with colored wheat lines.

In conclusion, the exploration of cereals and millets as essential and important sources of nutrients is critical in addressing global challenges such as food insecurity and hidden hunger. The studies featured in this Research Topic underscore the importance of biofortification and genetic enhancement to elevate the nutritional value of grains like pearl millet/sorghum/small millets. As we navigate the complexities of food processing and its impact on nutritional integrity, it becomes evident that a holistic approach is essential, considering genetic diversity, crop improvement, and nutrient bioavailability. The resurgence of interest in ancient crops like millets adds a valuable dimension to promoting dietary diversity and sustainable agriculture. Future areas of work should focus on refining biofortification strategies, advancing genomic approaches, and optimizing food processing techniques to preserve the nutritional benefits of cereals and millets. Additionally, promoting the integration of these nutrient-rich grains into mainstream diets is crucial for combating malnutrition and fostering a healthier, more resilient global food system.

## Author contributions

MM: Conceptualization, Writing – original draft. FJM: Writing – review & editing. OPG: Writing – review & editing. HKO: Writing – review & editing. KNG: Writing – review & editing.
